# “Access to pharmacy services is difficult in China”: a qualitative study from the perspective of transplant recipients to explore their expectations

**DOI:** 10.1186/s12913-024-10733-6

**Published:** 2024-03-27

**Authors:** Zhao Yin, Wan Zhang, Xuedong Jia, Xi Yang, Wenzhi Guo, Hang Fu, Shuzhang Du, Xiaojian Zhang, Fangying Si, Jun Li

**Affiliations:** 1 Institute for Hospital Management of Henan Province, Zhengzhou, China; 2 Department of Pharmacy, The First Affiliated Hospital of Zhengzhou University, No. 1, East Jian She Road, 450052 Zhengzhou, Henan Province China; 3 Department of Hepatobiliary and Pancreatic Surgery, The First Affiliated Hospital of Zhengzhou University, Zhengzhou, China

**Keywords:** Qualitative study, Organ transplant recipients, Pharmacy services, Perceptions and expectations

## Abstract

**Purpose:**

To gain an in-depth and comprehensive understanding of Chinese organ transplant recipients’ perceptions, expectations, and suggestions of pharmacy services to hospital pharmacists.

**Methods:**

This qualitative study was conducted in central China, from February to December 2020. Participants were collected with a purposive and snowball sampling method. Focus group discussions were conducted with organ transplant recipients and content analysis was applied to identify themes and subthemes.

**Results:**

21 recipients participated in the qualitative study. Four themes and thirteen subthemes were identified: (1) perceptions of clinical pharmacists and pharmacy services; (2) expectations for pharmacy service content; (3) expectations for pharmacy service form; and (4) difficulties as a special group.

**Conclusion:**

The pharmacy services provided by Chinese healthcare institutions are inadequate to meet the needs of organ transplant recipients. However, the acceptance and expectation of pharmacy services by transplant recipients are high. Therefore, China should learn from the experience of developed countries and focus on the actual needs of patients to establish a better pharmacy service system for organ transplantation.

**Supplementary Information:**

The online version contains supplementary material available at 10.1186/s12913-024-10733-6.

## Introduction

Organ transplantation is currently the most effective treatment for end-stage organ failure [1]. In the past few decades, the number of organ transplants in China has increased rapidly. Currently, China ranks 2nd in the world and 1st in Asia in terms of the number of organ transplants [2]. For organ transplant recipients, in most the cases, they need to take multiple medications for a lifetime [3]. It means that they need to face the complexities of adverse drug reactions and drug interactions for life, which is a great challenge. A comprehensive and high-quality pharmacy service system is essential to guarantee the survival and quality of life of transplant recipients. However, there are still deficiencies in the construction of pharmacy service systems for organ transplant recipients in developing countries such as China, and the pharmacy service needs of organ transplant patients are difficult to be adequately met [4]. This may be one of the significant reasons for the notable difference in the survival rates of organ transplant patients in China compared to developed countries such as the United States. Taking liver transplantation as an example, in 2020, the cumulative survival rates of recipients of citizen postmortem organ donation in China were 83.6% and 74.9% in the 1st and 3rd years postoperatively, respectively, while in the United States, they were 94.25% and 88.07% [5]. Although there are various factors influencing the survival rates of transplant recipients, such as donor-recipient matching, surgical technique, post-transplant ICU care, long-term care, and others; accurately identifying the pharmacy service needs of organ transplant patients to address the mismatch between supply and demand for organ transplant pharmacy services is an effective means for China and other developing countries to improve the survival rates of organ transplant recipients.

In the past decades, clinical pharmacy services for organ transplantation have developed rapidly in developed countries [6]. The designation of organ transplantation professional clinical pharmacists was established in 2018 by the Board of Pharmacy Specialties. In the USA, organ transplant pharmacists provide pharmacy services throughout the whole process from preoperative to postoperative transplantation, which meets the needs of both inpatients and outpatients [7]. The Canadian Society of Hospital Pharmacists has established the Transplant Pharmacists Network, which aims to promote “optimal clinical outcomes and patient-centered pharmacy practice” through information sharing. A systematic evaluation of 12 clinical studies showed that organ transplant pharmacists improved medication adherence, reduced morbidity, medication errors, and healthcare costs through patient counseling and education, optimization of medication regimens, and medication reconciliation [8]. Driven by the increasing demand for pharmacy services from organ transplant recipients, clinical pharmacy services for organ transplantation in China are gradually developing and have made some progress in recent years. However, the development of the organ transplantation clinical service system is relatively lagging behind in China [2]. One of the main manifestations is the lack of a comprehensive clinical pharmacy service system for organ transplant recipients. At present, only a few hospitals have opened organ transplantation pharmacy clinics, and specialized clinical pharmacists are even more lacking [2]. Most of the organ transplant clinical pharmacists are pharmacists who have received a full-time pharmacy or clinical pharmacy bachelor’s degree or above with chemical education and have been qualified by the 1-year full-release organ transplant professional or immune system drug standardization training held by Chinese Hospital Management Association or Chinese Medical Association. Domestic organ transplantation clinical pharmacists are relatively weak in medical knowledge and clinical experience [9]. Their service capabilities are insufficient, and their daily work is mostly focused on the determination of blood drug concentration and case reports of pharmacological monitoring of patients with post-transplantation infection [9]. Overall, the pharmacy service needs of organ transplant recipients in China have not been fulfilled. Therefore, it is necessary to conduct an exploratory and in-depth study designed from the demand-side perspective to explore the actual needs of organ transplant recipients for pharmacy services from their life constructs and personal experiences, and to provide empirical data for the construction of an ideal organ transplant pharmacy service system.

It is well known that pharmacy services are patient-centered and it is more relevant to recognize and evaluate pharmacy services from the patient’s perspective [10]. However, most of the available studies were conducted from healthcare practitioners’ perspective [11, 12]. In order to gain a more comprehensive understanding of the needs of patients, this study was designed to explore the experiences of transplant recipients with focus group discussions that are rich in interactive processes. In addition, most of the previous studies were cross-sectional studies based on questionnaires [4, 8]. Only a few studies have adopted a qualitative research design, using in-depth interviews to understand the pharmacy service needs of community residents [13], or refugees and asylum seekers [14]. From a methodological point of view, exploratory research from the perspective of transplant recipients is beneficial for us to delve deeper into their true ideas. By understanding their perceptions, needs and expectations of hospital pharmacists and pharmacy services, the present study aims to provide an objective basis for the construction of organ transplant pharmacy service systems in China and other developing countries.

## Methods

### Study design

This study was conducted as part of a larger research with the purpose of constructing of a life-cycle pharmacy service system for organ transplant recipients in central China and the current aim was to explore organ transplant recipients’ perceptions, needs, and recommendations for hospital pharmacists and pharmacy services. A qualitative research was conducted to achieve a comprehensive and in-depth understanding. The report was conducted under the guidance of the Consolidated Criteria for Reporting Qualitative Studies (COREQ) checklist, which was shown as Additional Table 1 [15].

The present study was approved by the Ethics Committee at The First Affiliated Hospital of Zhengzhou University (No.2022-KY-0735). Written informed consent forms were signed by all the participants.

### Recruitment

Participants were mainly liver or kidney transplant recipients in central China with the following inclusion criteria.

- Received organ transplantation for more than 1 year.
- Adults aged more than 18 years.
- Non-disabled patients with normal daily mobility.
- With sufficient communication and understanding skills.
- Willing to participate in the present study.

Maximum variation was also used for sex (male-female) and occupation (farmers, civil servants, medical professionals, teachers). The number of group discussions was determined by the saturation criterion (when additional interviews add no additional information) [16]. Children, patients with disabilities, and patients with poor communication skills had special pharmacy service needs and were excluded in the present study.

### Data collection

This study was conducted with semi-structured focus groups to gain insight into the feelings and expectations of the participants, a process that lasted from February 2020 to June 2020. A total of three focus group discussions were performed with seven participants in each group. The procedure was similar to our previous study [17]. Purposive sampling and snowball sampling were used to recruit participants. Researchers contacted patients directly by phone, WeChat, and email with recipients; or through clinicians who recommended suitable respondents according to the inclusion criteria; and through participant referrals (snowballing) to include new respondents. Prior to conducting formal interviews, we communicated with respondents about the purpose of the study and the interview format, and agreed on the interview time and location. Ultimately, all three interviews were scheduled in a quiet and comfortable conference room. The interview process ensured that no other people were disturbed and no clinicians were involved. The interviews were conducted by the researcher (ZY) and recorded by the researcher (WZ or XDJ), and the entire interview process was recorded. Based on the results of the literature study and the purpose of this study, the interview outline of this study mainly included the following aspects: the transplant recipients’ knowledge of hospital pharmacists and pharmacy services, their needs and expectations of pharmacy services, and the difficulties related to medication use encountered by the transplant recipients during the recovery process. The specific outline of the interview was shown in **Box 1**.

**Table:** 

Box 1. Questions used in the interview guide
Q1. How much do you know about hospital pharmacists and pharmacy services?
Q2. Have you ever received pharmacy services from pharmacists during your post-transplant medication use? What role do you think the pharmacist played in this process?
Q3. What are the problems you often encounter in the process of medication use?
Q4. In the process of communication with the pharmacist, what are the main issues when you communicate?
Q5. Please combine your own experience and talk specifically about what aspects of guidance or help you need from this pharmacist.

After the third group of focus group interviews, data saturation was reached and 21 transplant recipients finally participated in the first phase of the study. An overview of the demographic characteristics of the participants involved in the qualitative study are shown as Table 1.

**Table 1:** Demographic characteristics of all the participants

Demographic characteristics		Value
*Total number*, n	21	
*Sex, n*		
Female	6	
Male	15	
*Age, years*	49.24 ± 9.52	
*Level of education, n*		
Master’s degree	0	
Bachelor’s degree	9	
High school degree or below	12	
*Years of receiving organ transplant*	6.24 ± 7.21	
*Types of medications taken daily*		
Five types and below	12	
More than five types	9	

### Data analysis

Content analysis was used to analyze the interview data [18]. One researcher (ZY) verbatim transcribed the audio recordings within 24 h after the interview was completed, and two other researchers (XDJ and WZ) checked the transcripts. After confirming that the transcriptions were correct, the two researchers (ZY or WZ) independently carried out open-ended theme extraction to form the first draft of themes and subthemes. During this process, we used NVivo12 software to manage and analyze the data. Subsequently, the first draft of the themes and subthemes was discussed by the research team to propose changes to the theme structure and language expression. The final themes and subthemes were developed with the agreement of all, and appropriate representative quotations to present domains or sub-domains were selected.

### Trustworthy

The following measures were taken to ensure the trustworthiness of this study [19]: (1) the study team consisted of hospital pharmacists or social scientists with extensive experience in qualitative research, and close contact was maintained with the guiding experts to continuously resolve difficulties during the study; (2) after processing the transcribed interview data, the investigator confirmed with the participants that the results of the study were consistent with what the participants wanted to express; (3) the study was conducted in strict compliance with the operational procedures; (4) Self-reflexive reflections of the investigator: the principal investigator (ZY) has been conducting clinical pharmacy services in mainland China for many years and has extensive experience in pharmacy services; also, ZY completed an internship in the field of clinical pharmacy at the organ transplantation center of Yale New Haven Hospital as a visiting scholar. Therefore, ZY has a good understanding of organ transplant pharmacy services in both China and the United States, and can better explain the perceptions and expectations of organ transplant recipients, recognizing their recommendations as important references for future improvements in organ transplant pharmacy services.

## Findings

### Main findings from the qualitative study

In the qualitative study phase, 87 open codes were found out from the data. In the process of axial coding extra and unnecessary codes were omitted and 27 open codes were left. Finally, four main categories, thirteen sub-categories, and open codes were identified, which was shown as Table 2.

**Table 2:** Overview of the main categories and sub-categories and open codes

Main categories	Sub-categories	open codes
Theme 1. Perceptions of hospital pharmacists and pharmacy services	1.1 Inadequate perceptions of hospital pharmacists and pharmacy services	Lack of contact
		Only have the concept of a pharmacist
		Only know about pharmacist and no contact
		Don’t know what a pharmacist does
	1.2 Low recognition of clinical pharmacists and pharmacy services	Pharmacists only have the right to advise
		Pharmacists can not give professional guidance
Theme 2. Demand for pharmacy service content	2.1 Blood drug concentration monitoring	When and how to monitor the drug concentration
	2.2 Management of potential drug interactions	Drug interactions
	2.3 Guidance on the rational use of drugs	Consult the pharmacists for complications
	2.4 Management of adverse drug reactions	Deal with adverse drug reactions
		Inadequate advice from the doctor
	2.5 Popularization education of pharmacy knowledge	Lack of knowledge related to medicine
		Science popularization
		Popularize knowledge about hepatotoxicity induced by medicine
Theme 3. Expectations of the form of pharmacy services	3.1 Multidisciplinary and collaborative pharmacy services	Cooperation between physicians and pharmacists
		Combination between physicians and pharmacists
		Advice from the pharmacist
	3.2 Full life-cycle pharmacy service	Involvement of the pharmacist
	3.3 Convenient pharmacy services	Ask the pharmacists not the doctor for help Professional platform for inquires
		A phone or WeChat providing pharmacy services
		Lack of consultation way
Theme 4. Difficulties in rational drug use as a special group	4.1 Inadequate medical resources	Lack of pharmacy clinic
		General Practitioners can not answer me
	4.2 Medication adherence dilemma	Forget to take medicine
	4.3 Strong sense of confusion	Doubt about the doses of medicine
		Confusion with drug-food interaction

### Theme 1: perceptions of hospital pharmacists and pharmacy services

Participants expressed insufficient awareness and a low level of recognition of clinical pharmacists and pharmacy services. Most participants indicated that they were not clear about the content or the scope of pharmacists’ work and had not been exposed to hospital pharmacists or pharmacy services. In addition, a very small number of participants who had been exposed to hospital pharmacists had low recognition of hospital pharmacists. It was stated that the job of hospital pharmacists was only about drug dispensing, and pharmacists could not give patients professional guidance on rational drug use. A summary of the themes, subthemes, and representative quotations can be found in Table S1.

### Theme 2: demand for pharmacy service content

Participants claimed their needs for pharmacy services mainly included blood drug concentration monitoring, handling of potential drug-related effects, medication guidance, handling of adverse drug reactions, and popularization education of pharmacy knowledge. Firstly, participants paid the most attention to blood drug concentration monitoring and emphasized two aspects: (1) details of the preparation before the blood drug concentration test; (2) drug dose adjustment based on blood drug concentration monitoring results. Secondly, accompanied by other common diseases and therefore need to take other therapeutic drugs, they are particularly interested in learning about the interactions between these therapeutic drugs and the effects of their immunosuppressive drugs. In addition, participants expected pharmacists to provide advice on the rational use of over-the-counter medications. Meanwhile, most participants said that they were very concerned about the issue of adverse effects of post-transplantation therapeutic drugs. Finally, the participants hoped that hospital pharmacists could carry out popularization education of pharmacy knowledge to explain the doubts about daily drug use. Representative quotations can be found in Table S2.

### Theme 3: expectations of the form of pharmacy services

Patients’ expectations of pharmacy services are mainly reflected in the innovation of the form of pharmacy services, including the expectation that pharmacists and other medical personnel will cooperate as a team. Secondly, participants mentioned that the current model of pharmaceutical care was focused on the perioperative period and they wanted pharmacists to fully participate in postoperative medication management and provide a full range of pharmacy services. Their third expectation was about convenience. It was claimed that most regions in China have superior medical resources concentrated in large cities. Therefore, participants hope that medical institutions and pharmacists can make pharmacy services more convenient with the help of information technology such as professional apps, the WeChat public platform or WeChat group. A summary of theme 3, the subthemes, and representative quotations is shown as Table S3.

### Theme 4: difficulties in rational drug use as a special group

Interviewees emphasized that as a special group, their difficulties in rational medication use are mainly reflected in insufficient medical resources, medication adherence dilemmas, and a strong sense of confusion. Firstly, participants stated a lack of medical resources which was mainly reflected in the following aspects: (1) Due to the complexity of rational medication management for organ transplant recipients, general clinicians often refused to treat them; (2) There was a lack of professional organ transplant physicians and they are concentrated in large cities. So, it was difficult for transplant recipients to find organ transplant physicians to solve their problems; (3) the number of professional organ transplant pharmacists was even more lacking, and it was almost impossible for transplant recipients to access them. Secondly, the participant stated compliance-related dilemmas. Which was manifested in the following aspects: (1) difficulty in maintaining medication habits for a long time; (2) forgetting to take medication on time on special occasions, such as long-distance travel; (3) having doubts about long-term use of antiviral drugs and wanting to reduce or stop medication after a while. The last but not the least, transplant recipients had a lot of confusion about the rational use of medication, but these confusions were not resolved in time, which led to a strong feeling of helplessness. Representative quotations can be found in Table S4.

## Discussion

To our knowledge, the present study is one of the first to use a qualitative approach to explore organ transplant recipients’ perceptions, needs, and expectations of hospital pharmacists and pharmacy services in mainland China. This study provides an in-depth understanding of transplant recipients’ perceptions and potential acceptance of pharmacy services. At the same time, the findings provide directions for the future development of transplant pharmacy services.

The findings of the present qualitative study reveal a prevailing lack of awareness among patients regarding pharmacists and pharmacy services, aligning with the current status of delayed initiation and lagging advancements in clinical pharmacy within China [20]. Despite some progress, the clinical pharmacy system in China remains in its early or transitional stage [21]. Furthermore, due to socioeconomic disparities, such as uneven development between eastern and western regions, there exists a significant disparity in the distribution of pharmaceutical service resources across regions, urban and rural areas, and different levels of hospitals [22]. In underdeveloped regions, patients have limited direct interaction with pharmacists, resulting in lower levels of trust [23]. This could explain why many respondents in this study still exhibit a low level of recognition of the work of clinical pharmacists. However, the findings of this research also suggest a positive aspect: the majority of participants explicitly expressed their demand for and expectations of pharmaceutical services. This underscores that transplant recipients exhibit a high potential for accepting pharmacy services and harbor elevated expectations, thereby establishing a fundamental foundation for the development of organ transplant pharmacy services [7, 24]. In recent years, with the transition of pharmacy services to a “patient-centered” approach, the role of pharmacists in China has evolved from merely dispensing medications to directly providing services to patients [25]. This shift in focus has stimulated specific patient populations, such as organ transplant recipients, to express heightened demands and expectations for pharmaceutical services. Nevertheless, the current capabilities and service coverage of pharmacy services are yet to fully meet their needs. This observation indicates a directional guide for future development in this field.

The primary demand mentioned by participants in this study pertains to pharmaceutical services related to the monitoring of blood drug concentrations of immunosuppressants [26]. Immunosuppressive therapy is the cornerstone of successful transplantation in organ transplant patients [27]. In most cases, patients require lifelong immunosuppression, and with the need for transplant recipients to take multiple drugs simultaneously due to comorbidities, the management of adverse drug reactions and drug interactions becomes particularly critical [28, 29]. Therefore, monitoring of blood levels of immunosuppressive drugs has a significant impact on the prognosis of transplant recipients [26]. The findings of the present study showed that some patients do not know how to schedule their medication on the day of blood level monitoring. Pharmacists should use their professional knowledge to inform patients in detail about the specific time, dose, and precautions to take the medication [30]. In addition, unlike the general population, transplant recipients are more concerned about drug interactions between therapeutic drugs and immunosuppressants when they have mild illnesses such as colds and fevers [31]. Therefore, future organ transplant pharmacy service programs can focus on these areas and utilize their expertise to address the concerns of transplant recipients about the rational use of medications [32].

Over the past 20 years, the United States has gradually emphasized the importance of multidisciplinary pharmacy services in the postoperative medication management of organ transplant recipients [4]. In the United States, the multidisciplinary management team for organ transplant recipients includes clinicians, clinical pharmacists, nursing staff, dietitians, and counselors [7, 33]. This model also fits the practical needs of the participants in the current study. Therefore, a multidisciplinary collaborative pharmacy service model is an important direction for development [34]. In addition, participants in this study mentioned that access to appropriate medical care and medication after hospital discharge is a particular concern for transplant recipients [35]. However, the situation in China is that there is a lack of superior medical resources and they are very unevenly distributed [36]. In the future, we need to provide them with comprehensive and convenient pharmacy services through multimedia platforms such as telemedicine platforms, Internet hospitals or WeChat to improve the accessibility of advantageous pharmacy services [37].

There are several limitations of this study that are worth pointing out. First, the study population did not include hospital pharmacists in primary care, which is a notable regret. They will be studied in depth in our future study to provide a more comprehensive explanation of pharmacy services for organ transplant recipients. Meanwhile, the study participants were all from central China and the limited number of participants requires more caution to push the findings to a broader scope. Our next research plan will be to include more key informants using the Delphi method and to conduct a multicenter empirical study to further validate our findings. In addition, this study did not dig deeper into the reasons for low patient perceptions of clinical pharmacists, which we are addressing in another mixed-methods-based study we are conducting.

## Conclusions

This study provides an in-depth understanding of transplant recipients’ perceptions, needs and expectations of pharmacy services. In short, the pharmacy services provided by Chinese healthcare institutions are inadequate to meet the needs of organ transplant recipients. However, the acceptance and expectation of pharmacy services by transplant recipients are high. Therefore, China should learn from the experience of developed countries and focus on the actual needs of patients to establish a better pharmacy service system for organ transplantation. The results of this study also provide recommendations for the future development of organ transplant pharmacists, which mainly include providing more targeted pharmacy service programs, providing more convenient pharmacy service models, and improving the professional and technical skills and communication abilities of hospital pharmacists.

### Electronic supplementary material

Below is the link to the electronic supplementary material.

Supplementary Material 1

## Data Availability

Data are available on reasonable request. The thematic data that support the findings of this present study are available from the corresponding author on reasonable request.
