# Supplementary material for: “Access to pharmacy services is difficult in China”: a qualitative study from the perspective of transplant recipients to explore their expectations
Source: BMC Health Serv Res. 2024 Mar 27;24:387. doi: 10.1186/s12913-024-10733-6 (PMC10976827; doi:10.1186/s12913-024-10733-6)
Supplement: Supplementary file 1 — Supplementary Material 1 [file 12913_2024_10733_MOESM1_ESM.docx]

**Table S1: Theme 1 Perceptions of hospital pharmacists and pharmacy services**

|  | | |
| --- | --- | --- |
| Theme1 | | Perceptions of hospital pharmacists and pharmacy services |
| 1.1 | Inadequate perceptions of hospital pharmacists and pharmacy services | |
|  | From my experience I have not had any contact (with pharmacists), I have not actually had any contact with pharmacists since my transplantation, or I have not encountered any pharmacists. (G1P1) | |
|  | Practically all patients in the department do not have the concept of a pharmacist in their head. (G1P6) | |
|  | I only know about the profession (pharmacist), but I have not been exposed to this level, I have not come across (pharmacist). (G2P1) | |
|  | I just don't know what a pharmacist does. A pharmacist is one who dispenses medicine in a pharmacy, and as far as I can remember, I think a pharmacist is one who dispenses medicine. (G2P4) | |
| 1.2 | Low recognition of clinical pharmacists and pharmacy services | |
|  | If my test value (blood concentration) changes, I seek the pharmacist's opinion, you also answer, but at the end of the day, the doctor has the right to decide. Pharmacists only have the right to advise. (G2P4) | |
|  | I don't think pharmacists can give professional medication guidance to patients. (G2P1) | |

**Table S2: Theme 2 Demand for pharmacy service content**

| Theme 2 | Demand for pharmacy service content |
| --- | --- |
| 2.1 | Blood drug concentration monitoring |
|  | Every month when I have my blood drawn for blood levels, I am not sure when is the most appropriate time to take my immunosuppressants that day to minimize the impact on the results. (G3P3) |
| 2.2 | Management of potential drug interactions |
|  | For example, I have a cold and I want to find someone to ask if the therapeutic drugs have any effect on my anti-rejection medication. (G2P1) |
|  | Sometimes when you have a cold or another disease, can you take other drugs? (G3P1) |
|  | If there are other problems and I eat this usual immunosuppressant, then what is the conflict? Just find your pharmacist. (G3P2) |
| 2.3 | Guidance on the rational use of drugs |
|  | If you have a cold or diarrhea like this type (disease), you see which medicine I take and which I don't, ask the doctor, who may be in surgery at this time. Can I ask the pharmacist at this time? (G2P2) |
|  | Do we have a range of drugs for some common diseases, such as fever and cold, so that we can buy them in pharmacies and do not have to ask the doctor? (G3P3) |
| 2.4 | Management of adverse drug reactions |
|  | Post-operative medications often cause a variety of problems, such as mouth ulcers, high blood sugar, high blood lipids, diarrhea, some problems we contact the surgeon, they are also limited energy, pharmacists can help us? (G1P5) |
|  | You dare not (change the medication) yourself, and the doctor will not advise. When you can not tolerate the side effect, he will only suggest you to change it. (G2P4) |
| 2.5 | Popularization education of pharmacy knowledge |
|  | Although we have our clinicians to guide us, we are still not clear about the things (knowledge) related to medicine. (G3P1) |
|  | If I understand why I take so much medicine I will be more conscious, that is to say, Science popularization increases some science knowledge about postoperative medication, can make me better to cooperate. (G1P2) |
|  | I had surgery for hepatotoxicity, so I would like to call on pharmacists here to popularize this aspect for these special patients. (G3P2) |

**Table S3: Theme 3 Expectations of the form of pharmacy services**

| Theme 3 | Expectations of the form of pharmacy services |
| --- | --- |
| 3.1 | Multidisciplinary and collaborative pharmacy services |
|  | We hope that our team of physicians and pharmacists will collaborate to be a serious gatekeeper for us as operators in this quality of life. (G1P4) |
|  | There are clinicians and pharmacists combined to form a team to escort the whole process. (G1P3) |
|  | You should have at least one pharmacist following you no matter what the patient is, for all kinds of diseases, to give advice and make reference to the doctor. (G1P5) |
|  | If which indicators meet, the doctor to the pharmacist said in the future to the pharmacist management, if which is not normal, you come back to the doctor, so clearly tell us. (G2P4) |
| 3.2 | Full life-cycle pharmacy service |
|  | We hope that the pharmacist team will be more involved in our post-operative management (G1P3) |
|  | I think we should set up a post-operative department in the First Affiliated Hospital of Zheng University, in which the pharmacist team should be able to play a big role. (G2P2) |
| 3.3 | Convenient pharmacy services |
|  | I just give you a suggestion, you can have a professional what like APP or software or something, this is my post-operative which drugs can not eat, which things can not eat, which things are allowed to eat, like made this kind of thing the most suitable, a look to know. (G2P2) |
|  | Yes, it's best to have a professional platform, and once you check on it, I can eat this or not. (G2P2) |
|  | The first I think there is a public number of WeChat, the second I think there is a dedicated phone, I have A group and B group, otherwise a group words can not be added, more get a few groups can ah. (G2P1) |
|  | Or a place to register, or a phone or WeChat can be. (G2P4) |
|  | Most of them are in the group, there are questions are asked in the group. There is no other place to ask. (G1P4) |

| **Table S4: Theme 4 Difficulties in rational drug use as a special group** | |
| --- | --- |
| Theme 4 | Difficulties in rational drug use as a special group |
| 4.1 | Inadequate medical resources |
|  | In fact, patients want to ask the pharmacist, that is, does the pharmacist have a registration number, we just want to ask without a door. (G2P4) |
|  | The pharmacist is definitely more professional than us, right, that's for sure, and then you say who do you want to find, I can't find the doctor, then I find the pharmacist, right? (G2P2) |
|  | Usually, I have to talk to the doctor to buy drugs, but some doctors know a little bit, some of them are afraid to prescribe drugs for liver transplant. We have to find a transplant doctor, but they are hard to get an appointment, and there are no small hospitals. (G2P1) |
|  | I usually ask our doctor if I can eat this medicine, but he says ask your transplant doctor, we don't know. (G2P4) |
|  | You can only see the value of the examination, but in the middle of this missing several times, I do not know if it is right? There is no system. (G1P4) |
| 4.2 | Medication adherence dilemma |
|  | I missed a dose last week. I was probably busy with something and forgot about it, but now I think about it. (G1P5) |
|  | Sometimes I just forget to take my medication, like when I'm going on a trip and I've already put it on the table, but I still forget to bring it with me. Do pharmacists have any suggestions to help us take our medication on time? (G1P6) |
|  | Although I know I can't forget (forget to take my immunosuppressant). There are definitely times when you forget your medication, and there are times when anyone forgets. (G1P1) |
|  | But entecavir, ah, the doctor still let me take it for a long time, in fact, now my viral level is now almost undetectable, so why do I still have to take this antiviral drug. (G2P1) |
| 4.3 | Strong sense of confusion |
|  | For example, when I'm adjusting the medication, I'm especially concerned about whether I'm taking too much or too little, hoping that the smaller the amount of medication the better, without rejection, right? It's best to get it just right, but how do I know it's just right? Confused, right? (G3P2) |
|  | Should I take Chinese medicine or Western medicine? If you don't understand it, no one dares to eat it. So what should we do, confused? (G3P4) |
|  | Sometimes when you eat something you also consider, can eat can not eat. (G1P3) |
